# Diabetes Insipidus due to Metastases of Undiagnosed Lung Cancer: A Case Report from Syria

**DOI:** 10.1155/2023/1482675

**Published:** 2023-09-12

**Authors:** Ibrahim Alali, Alaa Al-Sarraj, Younes Kabalan

**Affiliations:** Endocrinology Department, Al-Assad University Hospital, Damascus University, Damascus, Syria

## Abstract

Pituitary metastases (PM) are extremely uncommon, accounting for less than 1% of all intracranial metastases. PM of an undiagnosed malignancy can commonly present with symptoms of hormonal deficiencies, central diabetes insipidus, and/or visual symptoms. Lung and breast malignancies are the most common cancers associated with PM. Despite advances in diagnostic and therapeutic options, the prognosis remains poor and is influenced by primary malignancy and treatment methods. We report a case of a patient with PM from lung cancer who had polyuria, polydipsia, and nonspecific symptoms. A full evaluation confirmed central diabetes insipidus, hypogonadism, and metastatic lung cancer. We also discuss the current literature on PM diagnosis and management, emphasizing the need for a comprehensive evaluation of all available data. This is the first case of PM reported from Syria, to our knowledge.

## 1. Introduction

Pituitary metastases (PM) are uncommon tumors that originate from cancers in other parts of the body and spread to the pituitary gland. They make up less than 1% of all intracranial metastases [1]. However, with the advancement of imaging techniques, more cases of PM are being detected. The most frequent primary cancers that cause PM are breast and lung cancers. PM can be asymptomatic or present with various symptoms, such as central diabetes insipidus (CDI), headache, and visual impairment [1–4].

We report a case of a patient who had fatigue, polyuria, polydipsia, and erectile dysfunction as manifestations of PM from lung cancer. We also discuss the diagnosis and management of PM in general. This is the first case of PM from Syria to be published in the literature. We aim to raise awareness about the possible association between lung cancer and PM, especially CDI, which may be overlooked or misdiagnosed in some regions of the world.

## 2. Case Presentation

A man aged 60 years presented to the endocrine clinic with polyuria, polydipsia, and erectile dysfunction. He had experienced these symptoms for two months and reported daily urine output of over 6 liters even when fasting, as well as excessive thirst. He received intranasal desmopressin without undergoing a water deprivation test, which alleviated his symptoms. He also complained of reduced libido and erectile dysfunction, in addition to weight loss, anorexia, chronic cough with sputum production, and fatigue. He denied having headaches or visual disturbances. He was admitted to the hospital for further evaluation.

The patient was married and had children. His medical history included unilateral renal atrophy. He was a retired smoker with a 60 pack-year history. He did not consume alcohol or illicit drug.

On physical examination, he had a temperature of 36°C, a pulse of 97 beats per minute, a blood pressure of 100/65 mm·Hg, and a respiratory rate of 20 breaths per minute. His body mass index (BMI) was 24 kg/m^2^. He looked well and had no skin hyperpigmentation. Lung auscultation revealed wheezes in the right middle zone. There was no hepatosplenomegaly or lymphadenopathy on palpation. The rest of the examination was unremarkable.

**Figure 1:**
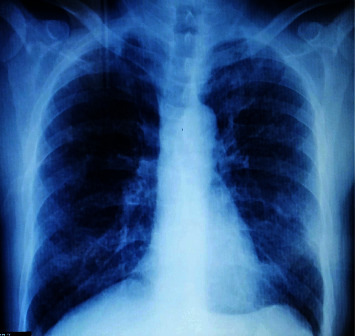
Plain PA chest X-ray, heart contour, diaphragmatic angles, and ribs are normal, bilateral hilar congestion, no obvious osteolytic lesions.

Initial blood and urine tests showed anemia and mild creatinine elevation (Table 1). A chest X-ray (posteroanterior view) demonstrated hilar congestion with emphysematous changes (Figure 1).

We performed a comprehensive evaluation for polyuria and anemia and found normal blood glucose and serum calcium levels (Table 1), high ferritin (1131 ng/ml; reference range 16.4–294 ng/ml), low folic acid (3.33 ng/ml; reference range 5.3–14.4 ng/ml) and vitamin B_12_ (238.1 pg/ml; reference range 240–900 pg/ml), and high lactate dehydrogenase (LDH) (667 U/L; reference range 240–480 U/L). A full workup to rule out possible malignancy was performed; upper endoscopy confirmed the presence of active chronic gastritis due to *Helicobacter pylori*. The Bence Jones test and prostate-specific antigen (PSA) were negative, and immune globulins were normal. Additionally, a tuberculin test and sputum staining with Zhiel-Neelsen stain were negative, and abdominal ultrasonography showed only left kidney atrophy.

We ruled out hyperglycemia, hypercalcemia, acute kidney injury, and mannitol use. Urine osmolality was less than 100 mOsm/kg H_2_O. We then performed a water deprivation test to diagnose diabetes insipidus (DI).

### 2.1. Water Deprivation Test

After eight hours of fasting and water restriction, we monitored blood pressure, weight, urine output, serum and urine osmolality hourly, and blood electrolytes every two hours. Six hours later, blood pressure dropped to 90/60 mmHg and two consecutive urine osmolality measurements were unchanged (110.65 and 145.19 mOsm/kg H_2_O). We stopped the test by giving 20 micrograms of intranasal desmopressin spray and measured urine osmolality after two hours at 330 mOsm/kg H_2_O, which was more than double the previous value, confirming central diabetes insipidus (CDI) (vasopressin deficiency).

To assess erectile dysfunction, we measured the testosterone level at 9 AM and found it to be low. We also tested other pituitary hormones and detected hypogonadotropic hypogonadism, hyperprolactinemia, low insulin-like growth factor-I (IGF-I), and normal adrenal and thyroid functions (Table 2).

**Figure 2:**
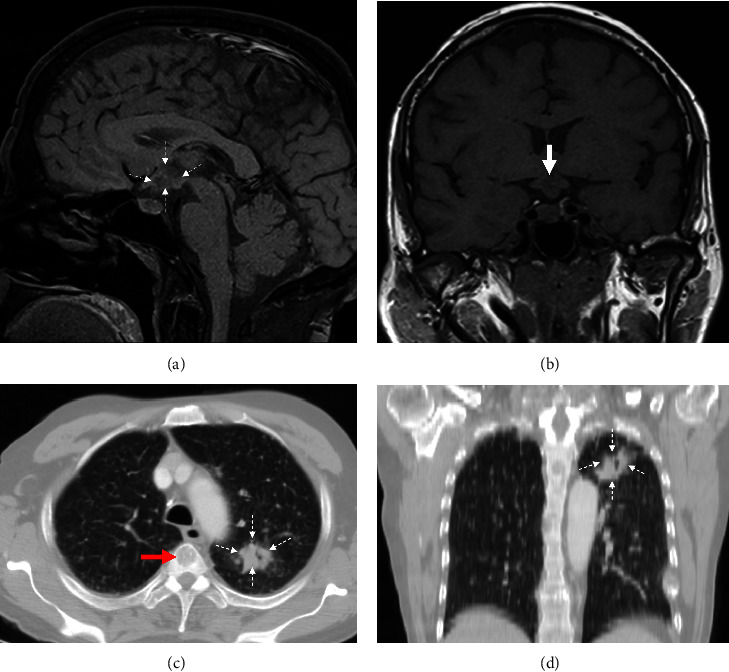
(a) The patient's sagittal MR image without contrast for the pituitary; the dashed arrowhead points to the hypothalamic mass. (b) Coronal image without contrast shows the optic chiasm and pituitary stalk infiltration. (c) Axial lung window shows the apical lung mass (the dashed arrowheads), and the red arrow points to an osteolytic lesion within the vertebral body. (d) Coronal lung window shows the apical lung mass (the dashed arrowheads).

A noncontrast pituitary MRI showed an infiltrative mass in front of the mammillary bodies that extended to the pituitary stalk and posterior pituitary with dimensions of 2.5 × 1.1 × 1 centimeters (Figures 2(a) and 2(b)). The radiologist also noted osteolytic lesions in the parietal bones indicative of metastases. The patient was uncooperative and could not complete a visual field examination.

A multislice computed tomography (MSCT) scan of the whole body revealed multiple osteolytic lesions and a 28 mm lobulated mass in the left lung apex that was not visible on the chest X-ray (Figures 2(c) and 2(d)). We obtained a CT-guided transthoracic biopsy, and the pathology report confirmed poorly differentiated non-small-cell lung cancer.

### 2.2. Patient Follow-Up

Due to the presence of distant metastases, the cancer was at an advanced stage. The oncology team assessed the patient and decided that no additional tests were needed. The patient received systemic chemotherapy after treating anemia and vitamin B_12_ deficiency. However, the patient died eight months after diagnosis, following multiple chemotherapy cycles and repeated hospitalizations with infections.

## 3. Discussion

The first case of pituitary metastases (PM) in the medical literature was reported by L. Benjamin in 1857 [5]. The prevalence of PM is estimated to be less than 1% of all intracranial metastases and surgically treated cases [1]. However, autopsy studies have shown a higher prevalence, ranging from 0.14% to 28% [5–7].

Many patients with PM have a history of cancer [1, 3]. However, some patients may present with hypothalamic or pituitary symptoms as the initial sign of an occult malignancy [1, 3, 7]. The proportion of such cases varies across different studies, from 7.8% to 40% [1–4, 7]. Interestingly, lung cancer was the most common primary tumor in these cases, as in our case [2, 3].

The primary tumors most frequently associated with PM are breast cancer in women and lung cancer in men [3, 8]. However, these two cancers account for more than two-thirds of all cases in many studies [1–3, 8, 10]. Other cancers such as kidney, colon, and less commonly liver cancer have also been reported in the literature [1, 2, 7–10].

In the absence of a history of malignancy, symptoms of pituitary involvement, and the primary tumor may overlap and delay the diagnosis [3]. PM does not have specific symptoms, but many series report headaches, visual disturbance, and ophthalmic nerve palsies [5–8]. Interestingly, our patient did not have any of these signs. Central diabetes insipidus (CDI) is a common feature of PM, ranging from 17 to 87% in different series [1–8], and it can be the first presentation of malignancy [6], as in our case. CDI is common in the presence of hypothalamic and stalk lesions since desmopressin is secreted from paraventricular and supraoptic nuclei in the hypothalamus and then transferred by neurons to the posterior pituitary [11]. Pituitary adenomas are more likely to cause anterior pituitary hormones disturbances than PM [3, 7], except for hyperprolactinemia, which occurs in 83% of PM cases [3]. However, the combination of CDI with or without anterior pituitary abnormality, an age over 50 years, and headache [3, 5, 7] are suggestive of PM, as seen in our case.

PM is challenging to identify radiologically when it affects the anterior pituitary and no primary malignancy is evident [2, 3]. In a series of pituitary stalk lesions, 25 (27%) of 92 cases had metastatic disease [12]. Moreover, 17.4% of cases with pituitary metastases had metastatic hypothalamic lesions [8]. In our case, MRI showed a normal anterior pituitary, a thickened pituitary stalk, an impaired optic chiasm, and a hypothalamic lesion suggestive of metastatic origin. This indicated the possibility of a primary cancer. Since a simple chest X-ray has a low sensitivity of 77–80% for detecting lung cancer in high-risk patients, more advanced radiological studies are required [13].

Histological diagnosis is the definitive method to confirm a diagnosis. However, factors such as clinical context, advanced stage of disease, lack of advanced diagnostic and treatment techniques, and limited resources influence the diagnostic and management plan. Habu et al. reported in their series that in 66% of published cases, histology was not verified and the diagnosis was presumed to be PM [8].

In many published cases, the prognosis is poor and depends on factors such as the prognosis of the primary tumor, the time between primary cancer diagnosis and PM, the presence of other metastatic sites besides the pituitary gland, and the availability of advanced therapeutic interventions [1, 14, 15].

In summary, we have presented a rare case of occult lung cancer that was diagnosed through its pituitary gland metastases. We wanted to highlight the importance of analyzing available clinical data in a resource-limited setting to establish the correct diagnosis without histologic confirmation.

## Figures and Tables

**Table 1:** Results of complete blood count and basic metabolic panel for both patients.

Test	Normal value	Patient's values
WBC (per mm^3^)	4.5–10.5 × 10^3^	13.4
Hb (g/dL)	11.5–13.5	9.6
PLT (per mm^3^)	150–450 × 10^3^	258
ESR (mm/hr1)	Up to 15	85
Urea (mg/dL)	10–50	52
Creatinine (mg/dL)	0.7–1.36	1.43
Glucose (mg/dL)	60–99	91
ALP (U/L)	Up to 270	253
ALT (U/L)	Up to 41	24
Calcium (mg/dL)	8.8–10.2	8.89
Phosphorus (mg/dL)	2.6–4.5	3.9
Sodium (mmol/L)	135–148	142
Potassium (mmol/L)	3.5–5	4.2
Chloride (mmol/L)	95–105	103
Serum osmolality (mOsm/kg H_2_O)	270–290	282.5
Urine osmolality (mOsm/kg H_2_O)	300–900	89
Urine analysis	—	Normal

WBC, white blood cells; Hb, hemoglobin; PLT, platelets; ESR, erythrocyte sedimentation rate; ALP, alkaline phosphatase; ALT, alanine aminotransferase.

**Table 2:** Results of hormonal evaluation along with reference range.

Hormone	Normal value	Patient's values
TSH (*µ*U/mL)	0.3–4.8	4.2
Free T_4_ (ng/dL)	0.8–1.8	0.86
IGF-I (ng/mL)	75–212	69.4
Testosterone (ng/mL)	2.27-10.3	0.249
LH (mU/L)	2–12	**1.63**
FSH (mU/L)	1.5–12.4	1.92
8 AM cortisol (*µ*g/dL)	5–25	35.91
Prolactin (ng/mL)	1.96–16.4	84.44

TSH, thyroid-stimulating hormone; IGF-I, insulin-like growth factor-I; LH, luteinizing hormone; FSH, follicular stimulating hormone.

## Data Availability

Data used in this study are available from the corresponding author upon a reasonable request.
